# Cancer Metabolism: Aspirations for the Coming Decade

**DOI:** 10.1101/cshperspect.a041555

**Published:** 2024-09-16

**Authors:** Ralph J. DeBerardinis, Karen H. Vousden, Navdeep S. Chandel

**Affiliations:** 1 Howard Hughes Medical Institute and Children’s Research Institute, University of Texas Southwestern Medical Center, Dallas, Texas, USA; 2 The Francis Crick Institute, London, UK; 3 Department of Medicine, Biochemistry and Molecular Genetics, Northwestern University Feinberg School of Medicine, Chicago, Illinois, USA

## Abstract

Fueled by technological and conceptual advancements over the past two decades, research in cancer metabolism has begun to answer questions dating back to the time of Otto Warburg. But as with most fields, new discoveries lead to new questions. This chapter outlines the emerging challenges that we predict will drive the next few decades of cancer metabolism research. These include developing a more realistic understanding of how metabolic activities are compartmentalized within cells, tissues and organs; how metabolic preferences in tumors evolve during cancer progression from nascent, pre-malignant lesions to advanced, metastatic disease; and most importantly, how we can best translate basic observations from pre-clinical models into novel therapies that benefit patients with cancer. With modern tools and an incredible amount of talent focusing on these problems, the upcoming decades should bring transformative discoveries.

Who knows how Otto Warburg would react to learning that his work on glycolysis and respiration launched an entire field of cancer biology, still accelerating after a century of discoveries. Warburg died in 1970. That was a pivotal time in cancer research, 11 years after Hungerford and Nowell described the Philadelphia chromosome, and a few short years before Varmus and Bishop reported the cellular origin of oncogenes that caused cancer. These discoveries in cancer genetics sidelined the “metabolic theory of cancer,” but only temporarily. Over the past few decades, advances in genetics, cell biology, molecular biology and biochemistry have become integrated into cancer metabolism research. This produced a sea change in how we think about metabolic reprogramming and how we study it, as indicated by the dozens of discoveries described in this book.

So what will the next few decades bring? Where are the current knowledge gaps, and how can we close them? Below we propose a few areas where progress has been bottlenecked by a lack of knowledge, technological capabilities, or both. Discovery in these areas could change the trajectory of the entire field.

## Metabolic compartmentation in cancer

Posters of metabolic pathways show the sequence of biochemical events connecting nutrients to end products, but they undersell the immense complexity of how these pathways operate in intact cells and tissues. In reality, metabolism is compartmentalized in various ways to enhance rapid flow from beginning to end, or to optimize flow by physically separating segments of the pathway from each other. Despite advances in the breadth and sensitivity of metabolic analysis, most assays still rely on bulk analysis of single tissues, making it difficult to probe spatial dimensions of metabolism. This is an important gap, because compartmentation contributes to flux regulation in many ways, including in cancer.

### Metabolic compartmentation within cells

The sequestration of enzymes and pathways into different membrane-bound organelles is the most basic and widely-studied aspect of metabolic compartmentation. Although enzyme localization within organelles has been known for decades, it has been difficult to obtain reliable assessments of the metabolic makeup of these compartments. New techniques enabling rapid harvest of mitochondria, lysosomes, peroxisomes, etc. have helped in this regard. Assessing metabolism in more porous organelles like the nucleus is still a challenge. This is relevant to cancer research because of the reliance of epigenetic enzymes on metabolites such as acetyl-CoA, S-adenosylmethionine and alpha-ketoglutarate, within the nucleus.

Some pathways are compartmentalized without the help of membrane-bound organelles, instead using the physical clustering of enzymes to facilitate substrate channeling and prevent intermediates from diffusing into the aqueous milieu^1^. The term “metabolon” refers to a form of pathway compartmentation defined by physical clustering of sequential enzymes in the same pathway to promote channeling and perhaps to enable metabolites arising from different sources (e.g. inside the cell vs. taken up from outside) to be processed differently^2^. The purinosome, composed of enzymes in de novo inosine monophosphate synthesis, is a classic metabolon that enables cluster channeling. This complex is assembled and disassembled during the cell cycle, presumably linking purine synthesis to genome replication^3^. The mechanisms allowing enzymes to participate in metabolon-like clusters are undefined, but it would be reasonable to explore whether growth-factor signaling cascades contribute to this process. This could open up new insights into the nature of metabolic flux regulation in both healthy tissues and cancer.

### Metabolic compartmentation among different cell types

The tumor microenvironment (TME) includes immune cells, stromal cells, endothelial cells and others in addition to the malignant cells. Current metabolomic technologies generally miss this complexity by using homogenized tissue samples that lump together all cell type. Research over the next decade needs to get past this kind of bulk averaging analysis. Ultimately we need to know the metabolic contributions of each cell type, how the metabolic properties of each cell type affect all the others, and how these metabolic interactions contribute to cancer progression. This will require significant technological advancements but should be worth the effort, because reductionist approaches have already identified examples of productive metabolic interactions among cell types in the TME. Co-culture experiments revealed that pancreatic stellate cells produce and secrete alanine, which is taken up and metabolized by pancreatic cancer cells^4^. In vivo, preventing stellate cells from releasing alanine reduced their ability to promote tumor growth. Axons from peripheral nerves can also release nutrients for consumption by malignant cells^5^. There are likely many more such examples, and we need better ways to directly probe the intact TME to systematically identify new aspects of metabolic crosstalk. Although further technical advances are needed, it is possible that mass spectrometry imaging (MSI) will eventually allow us to assess metabolic interactions between cell types, with resolution at the level of individual cells. There are also opportunities to use genetically-encoded metabolite sensors to study metabolic interactions in intact tissues.

### Metabolic compartmentation between tumor and distant organs

Tumor metabolism influences and is influenced by metabolism in the rest of the body. We are only in the early stages of understanding how these interactions affect cancer progression and health of the patient. Recent discoveries emphasize the potential for this area of research. Autophagy provides cell-autonomous metabolic advantages to cancer cells, but inactivating autophagy in the host reduces growth of transplanted tumors in mice. This indicates that some benefits of autophagy are extrinsic to the tumor^6^. One such benefit involves making nutrients systemically available to the tumor. Lack of hepatic autophagy causes the liver to release arginase-1. This depletes arginine from the bloodstream, inducing arginine starvation in tumors and reducing their growth^7^. Another example of organ-level metabolic compartmentation is the observation that glucose oxidation to CO_2_ flows in part through lactate. In this pathway, glucose is taken up and converted to lactate in one tissue, then lactate is secreted into the bloodstream, taken up elsewhere and oxidized to CO_2_ in the TCA cycle. This is relevant to cancer because the pyruvate oxidized in tumors is partially fed by circulating lactate, and this activity promotes metastasis in mice^8–10^. Advances in isotope tracing and computational flux analysis should permit the discovery of other mechanisms by which the contributions of multiple organs determine metabolism in tumors. There is also a pressing need to understand the biology of cachexia, which contributes to morbidity and mortality in many forms of cancer. This systemic phenotype likely involves compartmentalized metabolic activities in the tumor, liver, adipose tissue and muscle.

## The metabolic basis of cancer progression

Tumor metabolism is thought to evolve during progression from preneoplastic lesions to locally invasive tumors to lethal metastatic dissemination. We should seek to identify metabolic liabilities at every stage. An area where we currently know very little is in the nascent aspects of cancer progression: specifically, tumor initiation and growth into lesions large enough to come to clinical attention. We need to explore these processes, which may involve a combination of metabolic effects of somatic mutation, altered epigenetics, and interactions with the microenvironment. Understanding these metabolic influences could help explain how common co-morbidities such as obesity and diabetes increase the incidence of some kinds of cancer, and uncover interventions that could be used in at-risk individuals to reduce the number of patients who develop cancer. Mouse models will help, but the links between cancer and multifactorial metabolic conditions such as obesity and diabetes surely differ between humans and mice. We suggest that actionable discoveries will require intense clinical research specifically designed to uncover new aspects of pathophysiology in humans, followed by mechanistic work in mice and other experimental systems that recapitulate the most relevant components of the pathophysiology.

An orthogonal approach to studying the metabolic basis of cancer initiation is to focus on inborn errors of metabolism (IEMs) that cause cancer. These are rare monogenic diseases caused by germline mutations in genes encoding enzymes and other proteins involved in the metabolism. Because these are monogenic diseases, all aspects of the pathophysiology arise from a single defective node of the metabolic network. Some of these diseases enhance cancer risk, implying that certain metabolic perturbations are sufficient for cancer initiation in sensitive tissues^11^. This greatly simplifies how to think about the relationship between metabolism and cancer initiation. IEMs have already proven valuable in understanding the role of metabolic perturbation at the earliest stages of cancer. The seminal discovery that mutant isoforms of IDH1 and IDH2 produce *R*-2-hydroxyglutarate (*R*-2HG) in gliomas^12^ is an example of this. Among over 1,400 known IEMs, the only one associated with brain tumors is L-2-hydroxyglutaric aciduria, an autosomal recessive disease caused by defects in the enzyme that converts *S*-2HG into α-ketoglutarate. The fact that enantiomers of the same metabolite accumulate in both L-2-hydroxyglutaric aciduria and in tumors with IDH1/2 mutations strongly implied that elevation of *R*-2HG directly contributes to malignancy, as opposed to any other putative effects of mutant IDH1/2. This insight informed the development of drugs targeting mutant IDH1/2 and stimulated basic research into mechanisms of transformation.

Other IEMs promote cancer in different organs, particularly the liver. In Glycogen Storage Disease Type 1A, loss of glucose-6-phosphatase (encoded by *G6PC*) causes excessive glycogen accumulation, hypoglycemia, and hepatic tumors. A fraction of sporadic, adult-onset hepatocellular carcinomas also contain mutations in *G6PC*, raising the possibility that glycogen accumulation and/or associated metabolic disturbances are sufficient to cause cancer. Indeed, work from genetically-modified mice showed that glycogen induces liquid-liquid phase separation in hepatocytes in a manner that activates Yap and drives hepatocyte growth and tumorigenesis^13^.

Most cancer-related deaths result from tumors with acquired therapy resistance and/or metastasis to distant organs. Metabolic evolution in these later stages of progression is an active area of study and has been reviewed elsewhere^14^. We certainly need better ways to prevent tumors from reaching these late stages and to treat the ones that do. Several challenges cells must surmount along the metastatic cascade impose metabolic liabilities, and some are targetable in mice. These include the need to resist oxidative stress after escape from the primary tumor and the need to maintain metabolic flexibility during dormancy to use whatever nutrients are available at the metastatic site^15–17^. In both mice and patients, metastatic tumors activate the TCA cycle relative to tumors at the primary site^18,19^; the mechanistic basis for this shift is unclear but it occurs in multiple kinds of cancer, suggesting that it reflects a common metabolic demand during dissemination. Metabolic changes also accompany acquired therapy resistance because the cells emerging from cytotoxic therapy often share molecular properties with convergent metabolic preferences. For example, small cell lung cancers that relapse after platinum-etoposide therapy are enriched for *MYC* overexpression, and this induces metabolic liabilities that can be exploited to kill these otherwise highly resistant tumors^20,21^. The principle that relapsed and metastatic tumors are selected from pre-existing clones within heterogeneous primary tumors should make it possible to use lineage tracing techniques to identify the metabolic properties of cells destined to drive cancer progression.

## Next-generation approaches to metabolic therapy

Many attempts have been made over the past 20 years to target tumor metabolism in patients, so far with limited success. Mutant-selective IDH1/2 inhibitors have been effective in patients whose tumors contain the relevant mutations. But most other metabolic inhibitors have failed to produce clinical benefits beyond standard therapies, and some have caused dose-limiting, on-target toxicities. This has led to calls for more basic research before proceeding with additional interventional trials^22^.

There is no doubt that further exploration of fundamental mechanisms will help identify new pathways worth targeting and better explain how these activities support cancer progression. But regardless of how much preclinical work is carried out, we also need to re-think how we capitalize on information from model systems and how we deploy new investigational agents into clinical trials. The following are a few thoughts about approaches that could help increase the likelihood of success in clinical trials and reduce risks to patients unlikely to benefit from experimental agents.

First, there are untapped opportunities to incorporate metabolic analysis into patient stratification. Human tumor metabolism is incredibly heterogeneous, even among tumors from the same site. But most clinical trials studying metabolic inhibitors do not consider inter-patient metabolic heterogeneity. They choose tumor types that seem to share metabolic properties as a group rather than developing ways to assess metabolic properties within individual tumors. Surrogate biomarkers like gene expression are too often used to infer metabolic activity, rather than assessing metabolism directly, and proof-of-concept preclinical studies too often use models of questionable metabolic fidelity to tumors in patients. These uncertainties add up to a recipe for failure. Think of the experience with mutant IDH1/2 inhibitors as a counter-example, where mutations in IDH1/2 were used as enrollment biomarkers and levels of *R*-2HG could be used to assess pharmacodynamics. Other trials using metabolic inhibitors have nowhere near this level of clarity. Very few make a serious attempt to document which pathways are active in which tumors. Therefore, we cannot tell if a therapeutic failure means that the drug failed to inhibit the target, the tumor compensated by activating an alternative pathway, or simply that too many of the wrong patients got the drug. Yes, we need more basic science around metabolic targets. But in parallel, we should be developing techniques to reliably assess metabolic activity in tumors on a patient-by-patient basis, then learning how to integrate these approaches into clinical trials. Magnetic resonance spectroscopy, positron emission tomography, metabolomics and stable isotope tracing can all be used to assess tumor metabolism in individual patients. Perhaps better use of these techniques would improve the success rate of clinical trials.

Second, we can do more to develop rational combinations that include metabolic inhibitors. This is becoming essential because newer classes of therapies like targeted therapies and immunotherapies pervade modern clinical oncology. Demonstrating improvement over standard of care for a metabolic agent now often requires that it be tested in combination with other agents. Targeted therapies that inhibit growth factor signaling pathways block nutrient uptake and central carbon metabolism. Tumor cell metabolism is thought to contribute to immunosuppression within the TME. These considerations suggest ample opportunities to find contexts where metabolic blockade could synergize with existing drugs. Recent work with DRP-104 and similar molecules demonstrate this point^23,24^. These drugs are broadly-acting antagonists of enzymes that use glutamine as a substrate. Blockade of these enzymes suppresses tumor cell growth directly, but the drugs also enhance anti-tumor immunity by making the TME more hospitable to cytotoxic T cells. This results in potent combinatorial effects between DRP-104 and checkpoint inhibitors, a remarkable finding given that many metabolic pathways are thought to be shared between tumor cells and cytotoxic T cells.

Finally, we believe the time is right to thoroughly examine the preventative and therapeutic effects of dietary modification, and to define contexts where particular diets could be useful in patients. Given the immense capacity of the human body to tolerate dietary modification, this approach could lead to many different applications in many kinds of cancer. Recent work in mice has demonstrated the extent to which the diet impacts tumor dependence on metabolic pathways and modulates therapeutic responses^25,26^. Elimination of specific nutrients from the diet can be used in rational combination with metabolic inhibitors to suppress tumor growth in mice^27^. In addition to hypothesis-testing about dietary therapies in experimental models, we suggest that all clinical trials involving metabolic therapies should incorporate rigorous dietary records of all participants. This may uncover unexpected combinations that modify responses to the drug.

## Closing thoughts

The history of cancer metabolism research mirrors the progression of cancer itself. Initiated by Warburg, the field flourished for several decades before lying dormant, only to resurge together with new technologies and ideas, returning it to the leading edge of research. Discoveries from cancer metabolism have disseminated into many other areas of research, including stem cell biology, development, and immunity. The future of metabolism in cancer research is poised for significant advancements driven by an evolving understanding of metabolic reprogramming and its implications for tumor progression and therapy. Sophisticated animal models, advanced metabolic imaging techniques, and cooperative, multidisciplinary efforts will be crucial in translating findings from experimental models to clinical practice. This nuanced approach promises to reveal actionable metabolic vulnerabilities, leading to more effective and personalized cancer treatments.
